# Don’t be so naïve

**DOI:** 10.7554/eLife.103292

**Published:** 2024-10-25

**Authors:** Valerie Horsley, Aya Nassereddine

**Affiliations:** 1 Department of Molecular, Cellular, and Developmental Biology, Yale University New Haven United States; 2 Department of Dermatology, Yale School of Medicine New Haven United States

**Keywords:** cytoskeleton, human embryonic stem cells, naive pluripotency, stem cells, YAP, actin, Human

## Abstract

New evidence sheds light on actin regulation of pluripotency in human embryonic stem cells.

**Related research article** Meyer NP, Singh T, Kutys ML, Nystul TG, Barber DL. 2024. Arp2/3 complex activity enables nuclear YAP for naïve pluripotency of human embryonic stem cells. *eLife* **13**:e89725. doi: 10.7554/eLife.89725.

Before an embryo implants itself into the uterine wall, the stem cells it contains exist in a primitive ‘naïve’ pluripotent state from which they can develop into any cell type in the future body. After implantation, however, these cells transition to a primed pluripotent state where they exhibit more limited potential with each cell only being able to adopt a few predetermined fates. Once primed, the cells then differentiate into the multiple lineages of a developing embryo. It has become possible to partly influence these processes in the laboratory, with somatic cells being reprogrammed to become pluripotent, and certain pluripotent cells being induced to regain a naïve profile (Nichols and Smith, 2009). This has recently allowed for the development of exciting new methods that create human embryos ex utero (Pedroza et al., 2023; Oldak et al., 2023; Weatherbee et al., 2023).

Such work has been facilitated by a better understanding of the complex interplay of transcription factors, signaling pathways and epigenetic modifications that control pluripotency (Weinberger et al., 2016). Interestingly, mechanical cues that cells receive from their environment have also emerged as being modulated during differentiation or the exit from pluripotency, as well as being implicated in the reprogramming of somatic cells to pluripotent cells (Hogrebe et al., 2020; Rosowski et al., 2015; Gerecht et al., 2007; Hu et al., 2019). However, how mechanical signals regulate naïve pluripotency is not well understood.

Mechanical signals are relayed in part through cellular adhesions that anchor cells to each other and to their substrate. These processes typically involve the cytoskeleton, a complex and dynamic network of various protein filaments and associated molecules which, among other roles, help to transmit external forces through their connections to cellular adhesions and can generate mechanical forces via their interaction with motor proteins. Actin networks, for example, participate in how cells sense and exert mechanical forces on their environment (being closely connected to the outside of the cell via integrin-based cellular junctions) and, with the help of myosin and other molecular motors, regulate cell movement and cellular adhesion.

In pluripotent cells, actin organization has been shown to be essential for cell division, implantation and cell fate decisions in early murine embryos, and for driving the differentiation of human embryonic stem cells (or hESCs) in vitro (Yu et al., 2014; Hogrebe et al., 2020; Rosowski et al., 2015; Gerecht et al., 2007). When grown in 2D in the laboratory, hESCs at the outer colony edge exhibit robust actin cables, increased myosin activity and exert stronger forces on the underlying substrate; when induced to differentiate, these cells exit pluripotency first compared to interior cells (Rosowski et al., 2015). Yet, how cytoskeletal and mechanical cues control naïve and primed pluripotent states, and in particular the role that actin may play in these mechanisms, remains poorly understood. Now, in eLife, Diane Barber and colleagues at the University of California San Francisco – including Nathaniel Meyer as first author – report findings showing how actin dynamics are key to maintaining naïve hESCs (Figure 1; Meyer et al., 2024).

**Figure 1.**
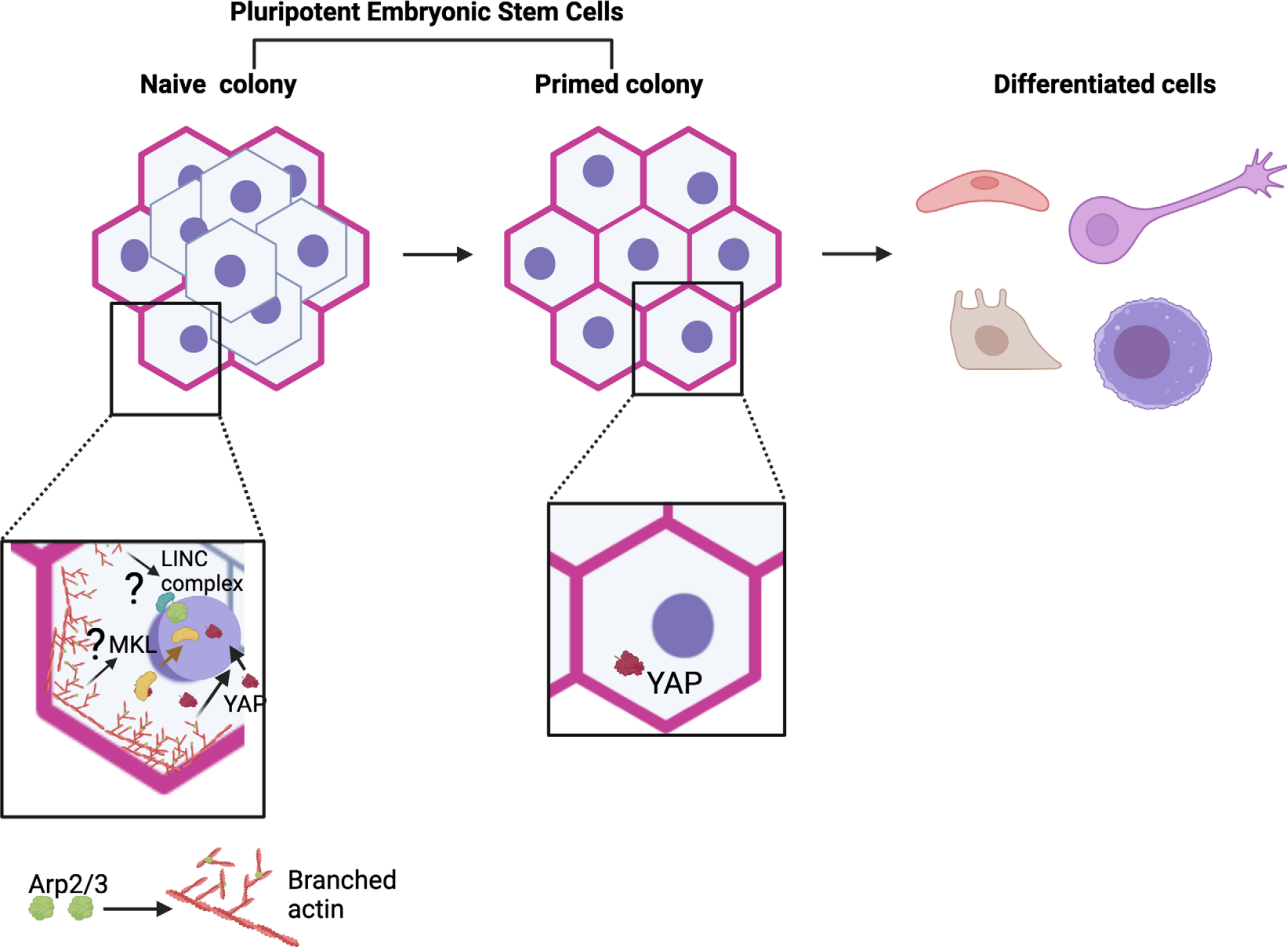
Model for actin and Arp2/3 regulation of naïve pluripotency. Pluripotent embryonic stem cells transition from a naïve to a primed state before differentiating. Meyer et al. show that colonies of naïve human embryonic stem cells (hESCs) exhibit a thick actin ring (pink) at their periphery, which is formed in part of branched actin filaments (red) generated by the Arp2/3 complex (green). This ring is absent in colonies of primed cells. Further experiments revealed that Arp2/3 activity is essential for the establishment of the naïve state (bottom left). This action is mediated by the relocation of the Yap protein (maroon structure and arrow) into the nucleus, where it can activate genes in the Hippo signalling pathway that are important for naïve pluripotency. This relocation relies on Arp2/3-driven changes in actin networks (red arrow); without it, cells cannot adopt a naïve state (bottom right). These results add to a body of work showing a role for Arp2/3 complexes in the control of pluripotency (bottom left), but their roles in naïve pluripotency have yet to be explored (question marks). For example, studies by Hu et al. revealed that MKL1 signaling (Megakaryoblastic Leukemia 1; yellow), which requires MKL1 to move into the nucleus (yellow arrow), represses reprogramming of somatic cells to pluripotency via Arp2/3-mediated processes. This involved the formation of actin networks that mechanically impact the nucleus (and therefore genome accessibility) through the LINC complex (blue and green), a molecular bridge connecting the cytoskeleton with the inside of the nucleus (Hu et al., 2019). Figure 1 was created with Biorender.com.

The team examined how actin was organized and functioned in hESCs artificially induced to transition from a primed to a naïve state (a process known as dedifferentiation). The experiments revealed that colonies of naïve hESCs, but not of primed hESCs, displayed a ring of thick actin bundles tethered to adherens junctions at their periphery. This resulted in differences in how the cells physically interacted with their underlying substrates. Primed cells exerted an elevated force which was evenly distributed throughout the colony, whereas naïve cells applied strong forces only at the colony periphery.

Further investigating the role of this actin ring, Meyer et al. used pharmacological approaches to inhibit the activity of the Arp2/3 protein complex, which is essential for the assembly of branching filaments of actin. Indeed, inhibition of Arp2/3 prevented the actin ring from forming during dedifferentiation, and it also stopped primed hESCs from adopting a naïve state. This manipulation also resulted in changes in the expression of genes in the Hippo signaling pathway, a molecular cascade involving the transcription coregulator Yap which can regulate the balance between stem cell self-renewal, tissue regeneration and organ size (Mo et al., 2014).

The experiments by Meyer et al. show that deactivating Arp2/3 led to Yap being blocked from relocating into the nucleus, where it can normally help turn on genes in the Hippo pathway. However, genetically modified cells that hold Yap in their nucleus regained the ability to dedifferentiate under Arp2/3 inhibition; interestingly, these colonies were also able to form the actin ring found in naïve hESCs. Taken together, these results suggest that Arp2/3 plays a key role in regulating pluripotency by driving changes in actin networks essential for Yap-mediated Hippo signalling (Figure 1). The findings also point to a bi-directional interplay, with Yap enabling the formation of actin structures found in the naïve state.

At first glance, Arp2/3 having a role in pluripotency may be surprising; the complex is often well-known for being involved in cell migration, a process that hESCs do not seem to actively participate (Papalazarou and Machesky, 2021). Arp2/3 can also regulate endocytosis, however, which can function in maintaining pluripotency (Narayana et al., 2019). While Meyer et al. did not explore whether endocytosis of key signaling receptors or cell adhesion receptors could help establish the naïve pluripotent state, other studies had already highlighted a role for Arp2/3 in pluripotency control.

Pluripotency is linked to changes in chromatin organization, with pluripotent cells having a more open chromatin landscape compared to somatic cells, for example (meaning that their genes are more accessible for activation or regulation). Arp2/3 has been shown to participate in biological processes that involve actin networks mechanically influencing the nucleus via a molecular bridge connecting the nuclear interior with the cytoskeleton; this, in turn, could influence chromatin accessibility (Figure 1; Hu et al., 2019). Arp2/3 can also be present in the nucleus, where it can bind to damaged chromatin sites and trigger the formation of actin filaments that alter chromatin organization and mobility – leading to speculations that nuclear Arp2/3 could function in pluripotency (Schrank et al., 2018; Tsopoulidis et al., 2019). Whether mechanical signaling or nuclear Arp2/3 controls naïve pluripotency will be an interesting area to explore in the future.

As for Yap, understanding its role in pluripotency is complicated by the fact that it can affect both the cell in which it is present and the neighboring cells. Work in somatic cells reprogrammed to become pluripotent suggests that Yap can block pluripotency in its host cell while also promoting this state in nearby cells by remodeling the extra-cellular environment (Hartman et al., 2020). The results by Meyer et al. support this model overall, showing that preventing Yap from performing its role drives cells to a naïve state and that it is also important for extracellular actin remodeling (such as the establishment of the actin ring). Evidence also suggests that this role may be important in the dedifferentiation of pluripotent cells, as Yap signaling can activate integrin and focal adhesion docking proteins to stabilize the actin cytoskeleton at the plasma membrane in other systems (Nardone et al., 2017).

By shedding light on the interaction between the Arp2/3 complex and the Hippo pathway, the work by Meyer et al. offers a promising avenue for advancing regenerative medicine – in particular by enhancing our understanding of how hESCs could be reprogrammed. Still, a key challenge lies in determining how these pathways can be fine-tuned for therapeutic applications without severely disrupting pluripotent and differentiation processes. Important questions also remain, such as the implication of microtubules, intermediate filaments and other cytoskeletal components in pluripotency transitions.
